# Samhd1 knockout mice: modeling retrovirus restriction in vivo

**DOI:** 10.1186/1742-4690-10-142

**Published:** 2013-11-20

**Authors:** Li Wu

**Affiliations:** 1 Center for Retrovirus Research, Department of Veterinary Bioscience, 1900 Coffey Road, Columbus, OH, 43210, USA; 2 Department of Microbial Infection and Immunity, Columbus, OH, 43210, USA; 3 Comprehensive Cancer Center, The Ohio State University, Columbus, OH, 43210, USA

## Abstract

The host dNTP hydrolase SAMHD1 acts as a viral restriction factor to inhibit the replication of several retroviruses and DNA viruses in non-cycling human immune cells. However, understanding the physiological role of mammalian SAMHD1 has been elusive due to the lack of an animal model. Two recent studies reported the generation of *samhd1* knockout mouse models for investigating the restriction of HIV-1 vectors and endogenous retroviruses *in vivo*. Both studies suggest that SAMHD1 is important for regulating the intracellular dNTP pool and the intrinsic immunity against retroviral infection, despite different outcomes of HIV-1 vector transduction in these mouse models. Here I discuss the significance of these new findings and the future directions in studying SAMHD1-mediated retroviral restriction.

## Introduction

The dNTP hydrolase SAMHD1 functions as a retroviral restriction factor in human non-cycling myeloid cells and resting CD4^+^ T-cells [1-7]. The molecular mechanisms by which SAMHD1 restricts retrovirus replication in non-cycling cells are not fully understood. Although SAMHD1-mediated HIV-1 restriction involves the depletion of the intracellular dNTP pool in non-cycling cells [4,5,7], recent studies revealed that phosphorylation of human SAMHD1 protein negatively regulates its HIV-1 restriction function [8-10]. The lack of SAMHD1 protein expression in humans due to *SAMHD1* gene mutations can result in a rare, but severe autoimmune disease called Aicardi-Goutieres syndrome (AGS). AGS is associated with elevated type I interferon (IFN) production and likely is due to an innate immune response to accumulated nucleic acids in the cell [11]. Despite the rapid research progress in understanding the molecular mechanisms of SAMHD1-mediated retroviral restriction, characterization of the physiological role of SAMHD1 *in vivo* has been elusive due to the lack of an animal model.

Generating a *samhd1* knockout (KO) mouse model can help elucidate the physiological functions of SAMHD1 *in vivo*[12,13]. Alternative splicing generates two isoforms of mouse SAMHD1 (mSAMHD1) proteins [12], which differ at the C-terminus and share about 72-74% identity with human SAMHD1 (hSAMHD1). mSAMHD1 possesses a similar dNTP hydrolase activity and also restricts *in vitro* HIV-1 infection as hSAMHD1 [4]. Both mSAMHD1 isoforms can block *in vitro* HIV-1 or SIV infections when they are ectopically expressed in a human monocytic cell line (U937) that does not express endogenous hSAMHD1 and is differentiated into non-cycling, macrophage-like cells [4]. Interestingly, two recent studies reported the generation of *samhd1* KO mouse models for studying retroviral restriction *in vivo,* while the effects of SAMHD1 deficiency on HIV-1 vector transduction in these mice appeared to be distinct [12,13]. The new findings and implications of these two studies are analyzed and discussed below.

## New findings and implications

### Generation of samhd1-null mice

Two studies by Rehwinkel *et al*. and Behrendt *et al*. generated conditional knockout strains in a mixed or a pure C57BL/6 background and obtained the *samhd1-*null mice by deleting exon 2 of the gene [12,13]. In both cases, homozygous *samhd1-*null mice had no detectable amount of SAMHD1 protein in bone marrow-derived dendritic cells (BM-DCs), primary mouse embryonic fibroblasts (MEFs), or splenocytes [12,13]. It is possible that these two *samhd1-*null mouse strains may have minor differences due to different approaches used in these studies [12,13]. Unlike the severe autoimmune disease in SAMHD1-deficient human patients with AGS [11], the *samhd1*-null mice were healthy beyond the age of 96 weeks [12] or up to 70 weeks [13], respectively. Neither SAMHD1-deficient mouse strain developed detectable pathology or autoimmunity [12,13], suggesting that mSAMHD1 function might be redundant or not directly involved in the innate immune responses to nucleic acids in mice. It also is conceivable that other triggers (such as cytokines, dysfunction of immune cells, or environmental factors) in human AGS patients may contribute to the disease pathogenesis in addition to the genetic deficiency of SAMHD1*.*

### Increased intracellular dNTP pool in samhd1-null mice

**Figure 1:**
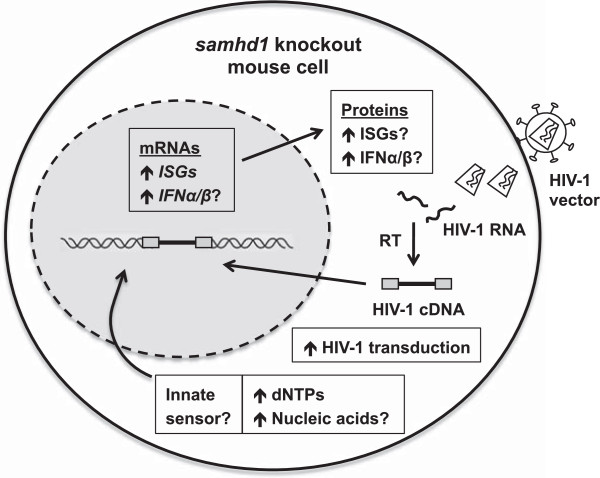
**HIV-1 infection and intrinsic immune response in SAMHD1-deficient mouse cells.** Intracellular dNTP concentrations are significantly increased in multiple cell types from *samhd1*-null mouse cells compared with control cells, indicating that mSAMHD1 acts as a dNTP hydrolase *in vivo* to reduce the intracellular dNTP pool. Spontaneous transcriptional induction of type I IFN-stimulated genes (ISGs) has been observed in several cell types and tissues from *samhd1*-null mice compared with wild-type mice. Therefore, mSAMHD1 might play a role in regulating the IFN signaling pathway *in vivo*, although the innate sensor that triggers the spontaneous ISG responses is unclear. Transduction of SAMHD1-deficient mice or derived cells with HIV-1 vectors is increased relative to wild-type mice or murine cells, which appears to be dependent on the affinity for dNTPs of the HIV-1 RT and the experimental conditions in these studies (refer to Table 1). RT, HIV-1 reverse transcriptase.

Using the *samhd1*-null mouse models, both studies confirmed that mSAMHD1 acts as a dNTP hydrolase *in vivo* to reduce intracellular dNTP concentrations in multiple cell types [12,13] (Figure 1). Compared with cells from wild-type mice, the intracellular dNTP concentrations were significantly increased in BM-DCs, bone marrow-derived macrophages (BM-DMs) [12,13], B-cells, T-cells, and MEFs [13] derived from *samhd1*^*-/-*^ mice. For example, the intracellular dTTP levels in BM-DCs from *samhd1*^*-/-*^ mice were quantified in both studies using different methods and showed an approximately 5-fold increase compared with those from wild-type mice [12,13]. Of note, the dTTP level in BM-DCs (0.5 μM) from wild-type mice reported by Rehwinkel *et al*. was 5-fold higher than that in human monocyte-derive DCs (~ 0.1 μM) from healthy donors [7]. This comparison suggests that dNTP levels in mouse cells are likely higher than those in human cells; however, it is difficult to directly compare these results since different approaches and cell types were used in independent experiments.

### Induction of type I IFN-stimulated genes in samhd1-null mice

Spontaneous and significant induction of mRNA expression of IFN-stimulated genes (ISGs) was observed in multiple cell types and tissues from *samhd1*-null mice compared with wild-type mice [12,13]. These results and the previous study by Rice *et al*. [11] suggest that SAMHD1 is a negative regulator in suppressing IFN-induced innate immune responses (Figure 1). Using a sensitive IFN bioassay, Rehwinkel *et al*. showed increased mRNA expression of several ISGs and the pro-inflammatory cytokine TNFα in spleens, macrophages and fibroblasts from *samhd1*-null mice compared with those from wild-type mice [12]. However, SAMHD1-deficient mice did not display detectable amounts of circulating IFN proteins or upregulation of ISG products in their sera or most tissues examined [12]. Using transcriptome sequencing, Behrendt *et al*. demonstrated that 123 ISGs were significantly up-regulated (85% of 141 genes showed signficiant up-regulation) in SAMHD1-deficient BM-DMs compared with those from control mice [13]. The up-regulation of ISG transcripts was dependent on IFNβ since crossing SAMHD1- and IFNβ-deficient mice prevented spontanous transcriptional activation of the ISGs [13]. Compared with wild-type mouse MEFs or BM-DMs, no difference of INFα induction was observed in SAMHD1-deficient cells treated with *in vitro*-transcribed RNA [12]. Notably, the undetectable type I IFN protein in the sera from SAMHD1-deficient mice [12] did not mirror the elevated levels of serum IFNα found in some human AGS patients [3,11], suggesting that SAMHD1 deficiency in mice is not sufficient to enhance the production or release of type I IFNs. Furthermore, similar levels of the induced INFα were detected in the sera of wild-type and SAMHD1-deficient mice infected with encephalomyocarditis virus, an RNA virus that can trigger INFα production [12]. These data suggest that mSAMHD1 deficiency is not sufficient to trigger IFN in response to nucleic acids or RNA virus infection in mice.

### Increased HIV-1 vector transduction in samhd1-null mice

Human blood monocytes and resting CD4^+^ T-cells from SAMHD1-deficient AGS patients are more susceptible to *in vitro* HIV-1 infection compared with normal cells [3,5,6], suggesting that hSAMHD1 contributes to HIV-1 restriction in non-cycling immune cells through an intrinsic mechanism. Despite significantly increased intracellular dNTP levels in SAMHD1-deficient mouse cells, Rehwinkel *et al*. did not observe any changes in single-cycle infection or transduction with HIV-1 or Moloney murine leukemia virus (Mo-MLV) vectors in *samhd1*-null mice or derived cells when compared with wild-type mice or cells [12]. Rehwinkel *et al*. interpreted that the lack of HIV-1 restriction in wild-type mouse cells was likely attributable to higher dNTP concentrations (~ 0.5 μM) than the K_M_ (~ 0.07 μM) of HIV-1 reverse transcriptase (RT). However, a ~5-fold increase in HIV-1 *in vitro* infection was evident in BM-DMs, BM-DCs and MEFs from *samhd1*-null mice using a vector expressing a mutant HIV-1 RT (V148I), which required higher levels of dNTPs relative to wild-type RT [12]. In line with this observation, Lahouassa *et al*. showed that the V148I RT mutant HIV-1 was approximately 6-fold less infectious in SAMHD1-sufficient human monocyte-derived macrophages compared with wild-type HIV-1 [4]. Furthermore, compared with wild-type mice, *in vivo* infection of *samhd1*-null mice with the mutant HIV-1 vector increased the viral transduction of splenocytes by ~8-fold and significantly enhanced transduction of myeloid and lymphoid cells as well as non-hematopoietic cells [12]. These results suggest that mSAMHD1 inhibits *in vivo* HIV-1 vector transduction by reducing the intracellular dNTP pool, which is in agreement with previous *in vitro* studies indicating that over-expressed mSAMHD1 blocks HIV-1 infection in differentiated human monocytic U937 cells [4].

In contrast to no effect on the transduction of *samhd1*-null mice with an HIV-1 vector expressing a wild-type RT reported by Rehwinkel *et al*. [12], Behrendt *et al*. used a different HIV-1 vector expressing a wild-type RT (Table 1) and demontrated a ~3- to 8-fold increase of *in vivo* transduction of splenocytes, CD4^+^ and CD8^+^ T-cells, B-cells, dendritic cells, and macrophages in *samhd1*-null mice compared with those in wild-type mice [13]. Moreover, *in vitro* infection of BM-DCs from *samhd1*-null mice using two different HIV-1 vectors expressing wild-type RT also showed a ~5-fold increase compared with control cells. Reconstitution of mSAMHD1 expression in BM-DCs from *samhd1*-null mice restored HIV-1 restriction, suggesting that mSAMHD1 contributes to the intrinsic anti-HIV-1 mechanisms *in vivo,* at least in part [13]. The discrepancy in results of HIV-1 transduction in these two strains of SAMHD1-deficient mice might be attributed to the different experimental approaches and conditions used in these studies [12,13] (Table 1). The viral infections and transductions in SAMHD1-deficient mice or derived cells reported in these two studies are compared in Table 1.

**Table 1:** **Comparison of viral infections of *samhd1 *** **KO and wild-type mice and derived cells**[12,13]

		**Rehwinkel *et al*. [**[12]**]**	**Behrendt *et al*. [**[13]**]**
*In vitro* HIV-1 vector infection of cells from *samhd1* KO mice compared with wild-type mice		• No effect on the infectivity of HIV-1 vector (wild-type RT) at 1–2 dpi* • 5-fold increase of HIV-1 RT mutant (V148I) vector infection in BM-DC, BM-DMs, and MEFs	• ~5-fold increased infectivity of HIV-1 vector (wild-type RT) in BM-DCs at 3 dpi* • Likely dependent on HIV-1 vector strains
*In vivo* HIV-1 vector transduction of *samhd1* KO mice compared with wild-type mice	Effects on HIV-1 vector transduction	• No effect on HIV-1 vector (wild-type RT) at 5–6 dpi* • 3-8-fold increase of HIV-1 RT mutant (V148I) in multiple cell types at 5–6 dpi*	• ~4-fold increased HIV-1 vector transduction (wild-type RT) in multiple cell types at 3 dpi*
	HIV-1 viral vector used	• pRRLsin.eGFP/ pCMVΔ8.2 vector (encoding Vif, Vpr, Vpu, and Nef) • HIV-1-based pCSGW/ p8.91 vector (no accessory genes)	• HIV-1_NL4-3_ based GFP reporter vector (pHR.CMVGFP/ pCMVDR8.91)
	Amount of HIV-1 vector injected	• Intravenously with 5 × 10^7^ or 1 × 10^8^ 293T cell infectious units	• Intravenously with 5 × 10^6^ viral particles
Exogenous Mo-MLV replication		No effect	ND^#^
Friend virus infection (a type of MLV)		ND^#^	No effect
Endogenous Mo-MLV replication or mouse retrotransposons		No effect	ND^#^
Encephalomyocarditis virus infection (a mouse RNA virus that induces IFNα)		No effect	ND^#^

*dpi, days post-infection; ^#^ND: not done.

### SAMHD1 does not affect endogenous retroviruses or retrotransposons in mice

It is conceivable that SAMHD1 may block endogenous retroviruses or retrotransposons by reducing the intracellular dNTP pool through its hydrolase activity. Rehwinkel *et al*. showed that SAMHD1 deficiency did not affect RNA levels of mouse long terminal repeat (LTR) retrotransposons and endogenous retroviruses (including MusD, IAP, Mu-ERV-L and m-poly-MLV) or the replication of endogenous Mo-MLV in BM-DCs and MEFs, or in lung and spleen tissues [12]. Therefore, mSAMHD1 appears to be independent of *in vivo* control of endogenous retroviruses and LTR retrotransposons in mice [12]. However, it is also possible that SAMHD1 may inhibit other retrotransposons or endogenous retroviruses in humans or mice. A recent study by Zhao *et al*. reported that diverse mammalian SAMHD1 proteins potently inhibited the non-LTR retrotransposon LINE-1 and LINE-1-mediated Alu/SVA retrotransposition in dividing human cell lines, suggesting that SAMHD1 may act as a cellular regulator of the conserved LINE-1 activity in mammals [14].

## Conclusions

The studies by Rehwinkel *et al*. and Behrendt *et al*. have established *samhd1*-null mouse models to better understand the physiological role of SAMHD1 *in vivo*[12,13]. Although SAMHD1 is not essential for mouse survival or development, it functions as a dNTP hydrolase *in vivo* and reduces the intracellular dNTP pool. The new findings from these studies suggest that mSAMHD1 can restrict lentiviruses *in vivo* through an evolutionarily conserved intrinsic mechanism, by decreasing intracellular dNTP concentrations [12,13]. Moreover, SAMHD1 deficiency in mice results in IFNβ-dependent transcriptional up-regulation of type I IFN-inducible genes and type I IFN signature in certain tissues and cell types [12,13], suggesting an important role of SAMHD1 in regulating innate immunity. The SAMHD1-deficient mouse model could be used as a potential tool to investigate the mechanisms of pathogenic type I IFN responses in autoimmune diseases in humans [12,13].

## Future directions

The molecular mechanisms underlying mSAMHD1-mediated restriction of HIV-1 *in vivo* are only starting to be investigated. Previous *in vitro* studies using cultured human myeloid cells and resting CD4^+^ T-cells have demonstrated that hSAMHD1 inhibits the accumulation of late reverse transcription products of HIV-1 [1,2,4-7], which is correlated with the reduced intracellular dNTP pool in these non-cycling cells [4,5,7]. It is important to know whether the increased HIV-1 gene expression in cells derived from *samhd1*-null mice is due to the increased levels of late reverse transcription products during HIV-1 cDNA synthesis, which has not been addressed in either study using SAMHD1-deficient mice [12,13]. However, current evidence suggests that decreasing the intracellular dNTP levels by SAMHD1 might be necessary, but not sufficient for HIV-1 restriction [8-10]. Recent studies indicate that cell differentiation-dependent phosphorylation of hSAMHD1 at residue T592 negatively regulates its anti-HIV-1 function [8-10], but not dNTPase activity [9,10]. It would be interesting to know whether phosphorylation of mSAMHD also affects its hydrolase activity and anti-retroviral function.

The cellular sources of type I IFN and the innate sensors that trigger the spontaneous IFN response in SAMHD1-deficient mice remain unclear [12,13]. Comparing cells types that produce type I IFNs in the various KO mouse models of AGS-associated genes and cells from human AGS patients can provide a means to identify the cellular sources of IFN production and the molecular mechanisms underlying spontaneous IFN induction in SAMHD1-deficient mice. These studies may help reveal the cellular and molecular mechanisms by which SAMHD1 modulates IFN and ISG expression.

SAMHD1-mediated HIV-1 restriction in human myeloid cells and resting CD4^+^ T-cells has been proposed as an immune evasion strategy for HIV-1 to avoid antiviral innate and adaptive immune responses and establish persistent infection [1,5,7]. SAMHD1 and Vpx interactions may contribute to the distinct pathogenic outcomes of HIV-1 and HIV-2 infection in humans. Additional *in vivo* studies using the macaque model and SIV infection as well as the humanized mouse model and HIV-1 infection could be useful to test this hypothesis.

Interestingly, recent genome-wide sequencing projects identified low frequencies of *samhd1* somatic mutations in several types of human cancer, including leukemia, lung adenocarcinoma, myeloma, medulloblastoma, glioblastoma, pancreatic, and breast and colorectal cancers (discussed in [12,15]). de Silva *et al.* reported significant down-regulation of SAMHD1 expression in peripheral blood mononuclear cells from T-cell lymphoma patients compared with those from health donors, which correlated with significantly increased levels of *samhd1* promoter methylation [15]. These studies suggest a potential role of SAMHD1 silencing in cancer development and the involvement of epigenetic mechanisms in regulating SAMHD1 expression. Therefore, in conjunction with other gene KO mouse models targeting tumor suppressors, the SAMHD1-deficient mouse models provide an important tool to explore the potential role of SAMHD1 in cancer.

## Abbreviations

SAMHD1: Sterile alpha motif domain- and HD domain-containing protein 1; hSAMHD1: Human SAMHD1; mSAMHD1: Mouse SAMHD1; AGS: Aicardi-Goutieres syndrome; KO: Knockout; BM-DCs: Bone marrow-derived dendritic cells; BM-DMs: Bone marrow-derived macrophages; MEFs: Mouse embryonic fibroblasts; HIV-1: Human immunodeficiency virus type 1; HIV-2: Human immunodeficiency virus type 2; Mo-MLV: Moloney murine leukemia virus; SIV: Simian immunodeficiency virus; dNTP: Deoxynucleoside triphosphate; dNTPase: dNTP triphosphohydrolase; IFN: Interferon; ISG: IFN-stimulated gene; LINE-1: Long interspersed elements 1.

## Competing interests

The author declares that he has no competing interests.
